# The Role of the Tumor Suppressor Gene Protein Tyrosine Phosphatase Gamma in Cancer

**DOI:** 10.3389/fcell.2021.768969

**Published:** 2022-01-05

**Authors:** Christian Boni, Claudio Sorio

**Affiliations:** Department of Medicine, General Pathology Division, University of Verona, Verona, Italy

**Keywords:** phophatase, PTPRG, cancer biology, tumor suppressor, pathway aberrant activation

## Abstract

Members of the Protein Tyrosine Phosphatase (PTPs) family are associated with growth regulation and cancer development. Acting as natural counterpart of tyrosine kinases (TKs), mainly involved in crucial signaling pathways such as regulation of cell cycle, proliferation, invasion and angiogenesis, they represent key parts of complex physiological homeostatic mechanisms. Protein tyrosine phosphatase gamma (PTPRG) is classified as a R5 of the receptor type (RPTPs) subfamily and is broadly expressed in various isoforms in different tissues. *PTPRG* is considered a tumor-suppressor gene (TSG) mapped on chromosome 3p14-21, a region frequently subject to loss of heterozygosity in various tumors. However, reported mechanisms of *PTPRG* downregulation include missense mutations, ncRNA gene regulation and epigenetic silencing by hypermethylation of CpG sites on promoter region causing loss of function of the gene product. Inactive forms or total loss of PTPRG protein have been described in sporadic and Lynch syndrome colorectal cancer, nasopharyngeal carcinoma, ovarian, breast, and lung cancers, gastric cancer or diseases affecting the hematopoietic compartment as Lymphoma and Leukemia. Noteworthy, in Central Nervous System (CNS) PTPRZ/PTPRG appears to be crucial in maintaining glioblastoma cell-related neuronal stemness, carving out a pathological functional role also in this tissue. In this review, we will summarize the current knowledge on the role of PTPRG in various human cancers.

## Introduction

**FIGURE 1:**
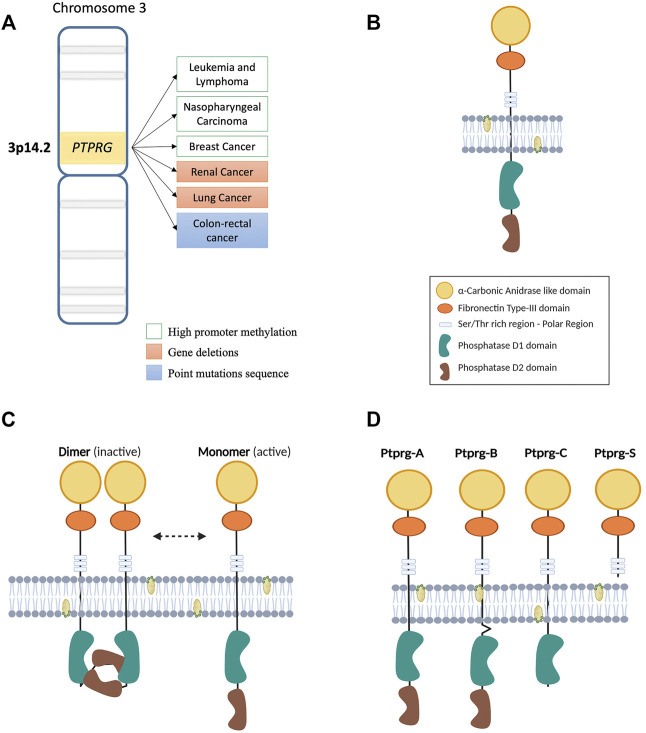
**(A)** Map of the region of human chromosome 3 where the PTPRG gene is located. On the side, the main genetic and epigenetic alterations involving the expression of PTPRG in various malignancies. **(B)** Illustration of the transmembrane structure of the PTPRG phosphatase. The different intra- and extra-membrane domains that form the protein are indicated in the legend on the side. **(C)** Schematic representation of the “head to toe” dimerization model of PTPRG phosphatase proposed by Barr et al., 2009. The inactive D2 domain of the first monomer of PTPRG interacts with the D1 domain of the second monomer making it blocked. The transition from inactive (dimer) to active (monomer) state can be performed using the technology of Trojan peptides (P1-WD) proposed by Montresor et al., 2021. Meanwhile the inhibition of phosphatase activity was performed using a specific inhibitor 3- (3,4-dichlorobenzylthio) thiophene-2-carboxylic acid in the work of Tomasello et al., 2020. **(D)** Illustration of the four different isoforms proposed for PTPRG.

Phosphorylation represents one of the best-characterized post-translational modifications, a form of structural change that can modify interactions and stability of the protein structure and modulate enzymatic activity. Since the discovery of proteins tyrosine kinase (PTKs) in the late 1970s, multiple evidences of the key pathogenetic role in cancer progression has emerged that led to countless investigations and discoveries on the regulatory mechanisms underlying signaling pathways governed by these critical enzyme (Hunter, 2009). On the other hand, the protein tyrosine phosphatase (PTPs) field developed with at least a 10 years delay and, being responsible for the removal of phosphate groups on tyrosine residues, they were viewed as a natural counter actors for oncogenic PTKs, becoming of great study interest as potential therapeutics target (Julien et al., 2011). A classic subdivision of the PTPs gene family is formed by receptors (RPTPs), particularly R1–R8 subgroups localized on cell membrane, and non-receptor (NRPTPs) including NR1–NR9 subgroups, localized predominantly in cellular interspaces such as cytoplasm (Alonso et al., 2004; Stoker, 2005). Other members of this large superfamily are represented by DSPs (Dual specificity phosphatases) and LMPs (Low molecular weight phosphatases). Despite a rather low specificity in *in vitro* assays, these enzymes possess a high substrate specificity in cells, mainly derived from the specific tissue distribution, restricted subcellular localization and from other post-translational modifications (e.g., phosphorylation) that regulate its functions (Tiganis and Bennett, 2007). Receptor-phosphatase usually are composed of a variable extracellular region combined with intracellular segment including phosphatase domains commonly shared in this superfamily. This union makes them suitable for coordinating both extracellular activities (e.g., cell-cell or cell-matrix adhesion) and intracellular signaling. Protein tyrosine phosphatase receptor gamma (PTPRG) belongs to the class of receptor PTPs similar to PTPRB/Z (Krueger and Saito, 1992), characterized by the presence of the homologous α-carbonic anhydrase like domain (CAH) and a Fibronectin type III domain in the N-terminal region (protein structure is represented in Figure 1B) (Barnea et al., 1993; Sorio et al., 1997). Beyond the membrane-spanning region, two highly conserved phosphatase domains (tandem domains) extend into the intracellular side. The catalytically active phosphatase domain D1 is proximal to the membrane, while the proximal C-terminal domain D2, lack the enzyme activity and is defined as pseudophosphatase domain. This inactive domain might be involved in stability, substrate specificity and binding of docking proteins (Barnea et al., 1993). Of note, the mutated form D1028A lacks phosphatase activity, rendering the PTPRG completely inactive (Zhang et al., 2012). Domains organization of the PTPRG molecule is fundamental for all tasks performed. Particularly the intracellular structure (ICD) plays a critical role in the regulation of phosphatase activity. Despite the receptor phosphatases dimerization was already known as a mechanism of inhibition (Jiang et al., 2000; Sonnenburg et al., 2003; Groen et al., 2008), also PTPRG on the cell membrane seems to self-associate forming a homodimer in a “head to toe” dimerization model. While further confirmations will be essential, the flexibility of the transmembrane domain of PTPRG would allow the inhibitory interaction, which is abolished with the mutation of several residues on the interface between both the D1 and D2 domains. Indeed, under normal dimerization conditions the PTPRG mutants have a higher catalytic activity than the wild type (Barr et al., 2009). Representation of the “head to toe” model of PTPRG is depicted in Figure 1C.

## PTPRG Functions in a Non-Neoplastic Context

Numerous cellular functions have been attributed to PTPRG due to its wide expression in different tissues (Vezzalini et al., 2007). The first specific functional data obtained from murine embryonic stem cells described a role in the differentiation of the hematopoietic compartment (Sorio et al., 1997), despite further analysis on another *Ptprg*-null mice model exhibited mild behavioral abnormalities without showing an obvious phenotype (Lamprianou et al., 2006; Zhang et al., 2012). A role in the regulation of blood flow/pressure and in vascular relaxation through intracellular Ca^2+^ response was described using the same murine model: under conditions of altered acid/base balance, such as low concentrations of [HCO^3-^], PTPRG-induced vasorelaxant effects, that were reduced in *Ptprg*-null mice (Boedtkjer et al., 2016; Hansen et al., 2020). Moreover, a similar process was described the proximal renal tubules, where the presence of PTPRG increased the reabsorption of HCO^3-^ (Zhou Y. et al., 2016). Both represent useful information to understand vascular conditions related to alteration of acid/base balance, such as stroke or heart attack, but also cancer cells and their microenvironment. Studies of PTPRG has focused on the central nervous system (CNS), which is characterized for the expression of various phosphatases (Lamprianou and Harroch, 2006). PTPRG is involved in the development of the spinal cord in chick (Hashemi et al., 2011), indeed its expression was detected in neuronal cells, more specifically in sensory neurons (e.g., pyramidal neurons in cortical layers II and V) and astrocyte (with higher expression in neuro-inflammation) while glial cells were slightly positive (Lamprianou et al., 2006; Lorenzetto et al., 2014). The lack of knowledge regarding the ligand for the PTPRG protein prompted Bouyain et al. to analyze some molecules belonging to the contactin family (CNTN), which are involved in cell adhesion and already known to be a PTPRZ/B ligand. CNTN1 binds to PTPRZ-expressing glial cells, increasing neurite growth and playing a role in CNS development (Peles et al., 1995). Similarly, biochemical and structural approaches have indicated an interaction between PTPRG and several members of this family (CNTN3 4, 5 and 6) (Bouyain and Watkins, 2010b). Instead, on cell surface, both *cis* and *trans* interactions between CNTN3 and PTPRG have been highlighted in neurons, hypothesizing a possible role in neuronal development, as already demonstrated for PTPRA (Bouyain and Watkins, 2010a; Nikolaienko et al., 2016). Increased PTPRG levels were reported in neurological and liver inflammation processes, suggesting a higher complexity level in the post-translational regulation of the PTPRG (Lorenzetto et al., 2014; Moratti et al., 2015). Particularly, Ptprg expression in liver has been indicated as a specific indicator of inflammation and a possible cause of insulin resistance (Brenachot et al., 2017). So far, pro-inflammatory factors such as LPS or IL-1β and TNFα were reported to upregulate *PTPRG* in astrocytoma cell line or astrocyte culture (Schumann et al., 1998; Lorenzetto et al., 2014) and also appear to associate to specific myeloid lineages, such as the differentiation of monocytes to dendritic cells (Lissandrini et al., 2006). Despite these examples (surely further roles in physiological context are expected to be uncovered) of an important role in normal cell physiology, the main data on PTPRG function derive from studies on its tumor suppressor role, since genetic alterations were found in numerous malignancies. For this reason, we mainly describe the available data on the mechanisms of expression and regulation of PTPRG activity in the context of neoplasia and potential clinical applications.

## Mechanisms Promoting PTPRG Silencing in Cancer

### Genomic and Epigenetic Processes

The first suggestion of an oncosuppressor role played by *PTPRG* is related to the non-random deletion in its chromosomal region in different types of carcinomas (Figure 1A) (LaForgia et al., 1991). Further analysis revealed loss of heterozygosity of the *PTPRG* locus in clear cell Renal Carcinomas (RCC), with no evidence of mutations in the 30 exons of the protein (Druck et al., 1995). Of note, this region was lately found to harbor the TS gene *FHIT* (Panagopoulos et al., 1996). On the other hand, observations on 12 microsatellites mapped on chromosome 3p12.2–21.1, specifically on marker D3S1239, showed a non-random loss both in NPC (nasopharyngeal carcinoma) cell lines and in three of seven (43%) of primary NPC samples (Cheung et al., 2008).

Studies on the genetic alteration of the phosphatases in human cancers evaluated the coding exons of 87 members of the PTPs genes superfamily. Examination of 3.3 Mb of sequences recognized somatic mutations affecting six PTP genes, including *PTPRG*. Specifically, the study of 157 colorectal cancers revealed eight cases (5%) harboring somatic mutations on the *PTPRG* gene (Wang et al., 2004). Detection of cancer alteration has shown that many mutations occurred in genes able to affect the DNA methylation status or controlling the chromatin structure. These analyses, performed with high-throughput technology, show that the epigenetic status of cancer can easily be a cause of the numerous mutations that occur in tumor cells (Shen and Laird, 2013; Klutstein et al., 2016). Exploring normal and cancerous colon mucosa using a CpG island microarray, specifically on a CpG-rich region in *PTPRG* intron 1, revealed that 17/18 colorectal carcinoma samples exhibited a fully methylated state. Furthermore, comparable data have also been identified in earlier carcinoma stages (e.g., serrated adenomas), and in Lynch syndrome-associated with colorectal cancers. Regardless of the methylation status and the tumor stages, no variation in *PTPRG* mRNA expression was detected. Nevertheless, the methylation of *PTPRG* intron 1, about 3 Kbp downstream of the transcription-starting site, reduced the binding of CTCF protein to the intron 1 sequence. (van Roon et al., 2011). The nuclear protein having 11 zinc-finger domains CTCF is highly conserved across species, from *drosophila* through mammalian radiation, and it enables vertebrates to regulate negatively and positively their transcription (Dunn and Davie, 2003; Kim et al., 2007). The reduced binding of CTCF with the intron 1 of *PTPRG* sequence could weaken the formation of chromatin regulatory structures essential for the expression of distant genes (van Roon et al., 2011). Additionally, high levels of methylation have been found in chronic myeloid leukemia (CML) patients (Ismail et al., 2020). Instead, *PTPRG* promoter methylation was regularly associated with decreased protein expression and has been reported in several malignancies. Shu et al. described a reduction of *PTPRG* mRNA in breast cancer cell lines MCF-7 and SK-Br-3, compared to a non-cancerous cell line MCF-10A. Small *PTPRG* mRNA values were identified in cancer cell lines that matched with the methylation pattern evaluated using TaqI restriction enzyme by COBRA assay (Shu et al., 2010). Similarly, hypermethylated status of the *PTPRG* promoter was characterized in NPC cell lines by methylation-specific PCR. Expanding the analysis to seven human NPC biopsies, paired with the counterpart of normal tissue showed *PTPRG* methylated alleles only in tumor tissues (Cheung et al., 2008). Similar alterations were reported in hematological malignancies, such as in acute lymphoblastic leukemia (ALL) and in cutaneous T-cell Lymphoma patients (Chatterton et al., 2014; Stevenson et al., 2014). A genome-wide investigation of promoter CpG islands identified several membrane-bound tyrosine phosphatases frequently methylated, including *PTPRG* (van Doorn et al., 2005; Kuang et al., 2008). This initial screening prompted Stevenson et al. to investigate the methylation status of 22 leukemic cell lines, demonstrating a strong promoter methylation of *PTPRO* phosphatase, while *PTPRG* and others showed variable patterns between myeloid and lymphoid cell lines. Significantly, higher methylation levels were also identified in 57 ALL patient samples, with a *PTPRG* promoter methylation rate of 63% (Stevenson et al., 2014). Concurrently, direct correlation was established between *PTPRG* methylation (both CpG in the promoter and in gene body) and *RAS*-mutated phenotype in ALL childhood patients (Chatterton et al., 2014; Xiao et al., 2014). Indeed, the KRAS-induced transcription factor RREB1 was shown to be able to bind a *RAS-Responsive Element* (R.R.E.) on the *PTPRG* promoter region. This feature emphasizes a KRAS-induced modulation of the phosphatase expression, especially after treatment with a demethylating agent, emphasizing the relevance of epigenetic regulation (Xiao et al., 2014).

The analysis of *PTPRG* expression led to its characterization also in the myeloid lineage (Lissandrini et al., 2006) and in myeloproliferative diseases (Della Peruta et al., 2010). Recently our laboratory demonstrated an intense correlation between *DNMT-1* and *3b* expression, two DNA methyl-transferases cooperating in tumor suppressor genes silencing (Rhee et al., 2002), and the reduction of *PTPRG* expression in CML. These two methyl-transferases were found highly expressed in CML cell and chromatin immunoprecipitation revealed the engagement of DNMT-1 to the PTPRG promoter sequence (Tomasello et al., 2020). Furthermore, recent results have highlighted a high frequency of CpG island methylation in CML patients compared with control group. Interestingly, hypermethylation of CpG islands in *PTPRG* intron 1 was identified in a group of patients that failed the tyrosine kinase inhibitors (TKIs) response compared to a newly diagnosed one (Ismail et al., 2020). In almost all studies performed on the methylation of *PTPRG* promoter, treatment with demethylating agents (including 5-azacytidine) restored the expression of *PTPRG* (van Doorn et al., 2005; Cheung et al., 2008; Della Peruta et al., 2010; Shu et al., 2010; Stevenson et al., 2014; Xiao et al., 2014; Tomasello et al., 2020). This results support how epigenetic silencing represent a general mechanism to modulate *PTPRG* expression, especially in leukemia (Figure 1A).

### Post-Transcriptional and Post-translational Regulation

PTPRG protein is known to undergo some specific processing: four different isoforms have been described as alternative splicing in rat brain cells (Figure 1D) (Shintani et al., 1997). Besides the classic whole structure of the phosphatase, the truncated form of the extracellular domain seems to be of particular relevance. Indeed, increasingly evidence emphasizes a role in several tissues under inflammatory state (Lorenzetto et al., 2014; Moratti et al., 2015; Jiang et al., 2020). As previously discussed, PTPRG is regulated also in the context of myeloid cell differentiation (Lissandrini et al., 2006). In addition, the entire active form of PTPRG phosphatase protects breast cancer cell lines from the increase in cell growth and proliferation induced by estradiol-17β and zeranol, both of which may induce an estrogenic response (Liu et al., 2004). Considering this, Wang et al. found a lower amount of *PTPRG* mRNA in breast cancer tissues compared to cells from healthy tissues. Subsequently, they investigated the role of conjugated linoleic acids, natural compounds protecting breast cancer cells from estrogenic proliferative effects. The t10, c12-CLA and t9, c11-CLA were shown to enhance *PTPRG* expression in breast cancer cell lines but also in human cancerous tissues. This condition occurs mainly in epithelial cells, with no effects on stromal cells, indicating selective *PTPRG* regulation of these compounds and their antitumor role in breast cancer (Wang et al., 2006).

Forefront genomic techniques afforded the study of non-coding RNA forms, a massive component in the human genome that participates in the transcriptome regulation. Alteration of the levels of these critical ncRNAs have been shown to promote tumorigenesis (Goodall and Wickramasinghe, 2021). *PTPRG* expression is regulated by different ncRNAs, classified according to structurally different molecules and their biological roles exerted (Table 1). Several microRNAs (miRNAs) composed by 18–25 nucleotides might induces mRNA modulation by guiding gene expression through the binding to the 3’ UTR region of the mRNA (Ha and Kim, 2014). Altered expression of several miRNAs, such as those belonging to the polycistronic miR-17–92 cluster, has been associated with tumorigenesis (Hong et al., 2010). Liu et al. analyzed the role of *PTPRG* in human breast cancer, confirming the dramatic reduction of PTPRG protein compared to healthy human tissue. Post-transcriptional regulation of *PTPRG* has been indicated as a consequence of increased levels of miR-19b only in cancer tissues. Indeed, treatment with an anti-miR-19b subsequently restored PTPRG protein expression levels. Finally, a *PTPRG*-specific siRNA simulates the phosphatase protein reduction increasing the tumorigenic capability of cancer cell lines, confirming the tumor suppressor role played by PTPRG in human breast cancer (Liu et al., 2016). Similarly, miR-141 belonging to the miR-200 family affects the expression of *PTPRG* in renal tissue. Specifically, the effect demonstrated by Newbury et al. reveals the increased value of miR-141 in acute kidney injury (AKI). By inducing miR-141 and causing a similar cell oxidative stress (H_2_O_2_) the reduction of PTPRG expression was achieved. MiR-141 increased cell death and decreased viability in PTEC cells, obtaining the same results by siRNA transfection against *PTPRG* (Newbury et al., 2021).

**TABLE 1:** ncRNA targeting *PTPRG* in disease processes.

ncRNA	Tissues source	Expression	References
mir-19b	Breast	Upregulation	Liu et al. (2016)
mir-567	Lung	Upregulation	Yu et al. (2019)
cMras	Lung	Downregulation	Yu et al. (2019)
mir-141	Kidney	Upregulation	Newbury et al. (2021)
lncRNA-AS1	Brest, Bone, others	Alteration	(Zhao et al., 2014; Iranpour et al., 2016; Ge et al., 2021)

CircRNAs are a novel class of the untranslated RNA, usually used as initiation/progression diseases markers, characterized by a special circular structure and a higher forbearance to exonucleases (Meng et al., 2017). Recent evidence in lung cancer indicating a crucial role for reduced levels of has_circ_100,395, a circRNA that operate as a sponge for miR-1228 that was involved in cancer development (Chen et al., 2018). In addition, the study involving another circRNA hsa_circ_0067512 (cMras) indicated its downregulation in human lung adenocarcinoma (LUAD) tissues and LUAD cell lines. Since circRNAs are known to control mRNA functions, Yu et al. identified a potential regulatory process enabling cMras/miR-567 to modulate *PTPRG* expression. Particularly, the reduction of cMras in LUAD left the miR-567 free to bind the 3’ UTR of *PTPRG* mRNA, reducing its expression. The effects obtained by this cancer mechanism reflect the increase of proliferation/migration in lung cancer cells. Finally, a worse prognosis was revealed in LUAD patients with low levels of PTPRG expression suggesting a protective role by phosphatase in this cancer (Yu et al., 2019). The ncRNAs governing PTPRG-expression in disease processes are shown in Table 1.

Although we know only a little fraction of functional lncRNAs to date, these transcribed ncRNA molecules longer than 200 nt have been shown to modulate each level of gene expression. Post-transcriptional gene silencing can occur through the category of lncRNA antisense, such as PTPRG-AS1 (Faghihi and Wahlestedt, 2009; Wang and Chang, 2011). PTPRG-AS1 expression has been considered as an oncogenic factor in several cancers. Additionally, *PTPRG* gene expression may be affected by high manifestation of PTPRG-AS1 affecting its TSG functions. These evidences were associated with increased survival of breast cancer patients who had a specific pattern of three lncRNAs, including reduced levels of PTPRG-AS1 (Zhao et al., 2014; Iranpour et al., 2016) (Table 1).

## PTPRG Interacts With Characteristic Oncogenes in Specific Cancer Types

### Leukemia and Lymphomas

**FIGURE 2:**
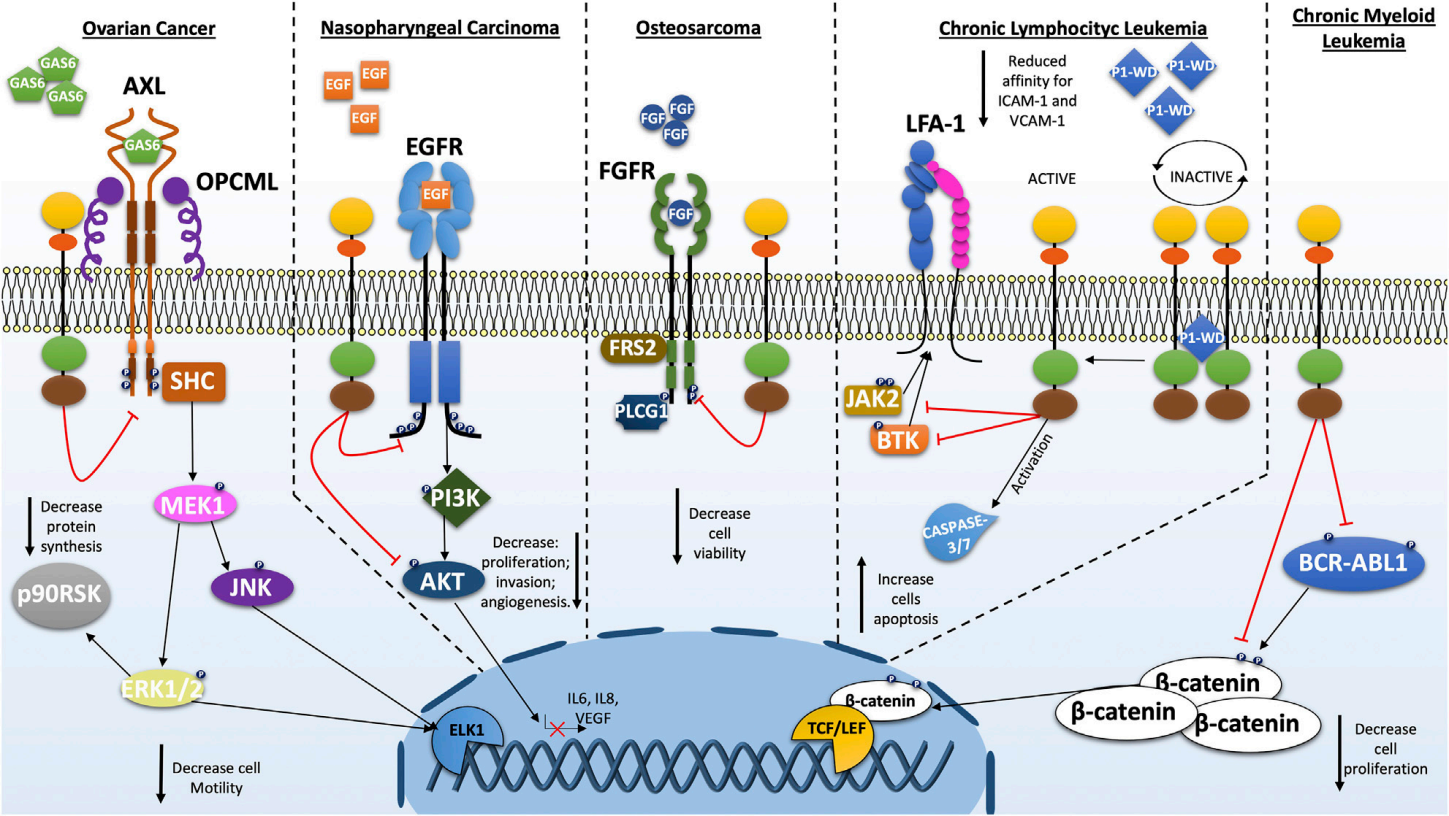
Schematic representations of the cellular pathways most investigated on the role of PTPRG in various types of cancer. As a tumor suppressor, PTPRG regulates several oncogenes such as BCR-ABL1, EGFR, FGFR, and others. The alteration of PTPRG emphasizes some cancer-associated cellular characteristics such as proliferation, motility and invasion.

Various evidence reflects the critical role of *PTPRG* in bone marrow and peripheral blood malignancies. Investigations carried out in the various leukemia and lymphoma subgroups report a significant involvement of *PTPRG* underlined by multiple data obtained on the epigenetic mechanisms affecting this gene (van Doorn et al., 2005; Chatterton et al., 2014; Stevenson et al., 2014; Xiao et al., 2014). In this context, PTPRG has been shown to negatively modify the ERK1/2 kinase phosphorylation in cell line model expressing mutant *KRAS* able to alter several signal cascades including AKT, ERK1/2, IκB-α, JNK and p38 MAPK (Xiao et al., 2014). Chronic lymphocytic leukemia (CLL) was defined as B lymphocytes accumulation, both in primary and secondary lymphoid organs, which are characterized by extended cell life (Pangalis et al., 2002). Laudanna group reported that PTPRG was found involved in the regulation of the BTK/JAK2 axis in the CXCR4-and BCR-triggered integrin activation (Mirenda et al., 2015; Montresor et al., 2018). Trojan peptide-mediated activation of PTPRG (P1-WD) demonstrated the ability to reduce both JAK2 and BTK phosphorylation by producing a strong reduction in the integrin-mediated adhesion capability of healthy and leukemic B-lymphocytes (Figure 2). Moreover, activated PTPRG was able to induce the apoptotic process as intensely as the BTK inhibitor Ibrutinib, specifically in CLL and not in healthy B-lymphocytes. These results were also confirmed by the use of the whole D1 catalytic domain engineered in a membrane permeable form (TAT-ICD) (Montresor et al., 2021).

The first hematological malignancy where a key role of *PTPRG* was discovered is the Ph^+^ chronic myeloid leukemia (CML). Della Peruta et al. reported how the tyrosine phosphorylation of BCR-ABL1, CRKL and STAT5 were decreased in *PTPRG*-transfected K562 and reported downregulation of PTPRG mRNA and protein in CML patients (Della Peruta et al., 2010). This type of result was independently replicated confirming the inhibitory activity of PTPRG on BCR-ABL1 protein and on substrates influenced by itself such as ERK1/2 kinase (Drube et al., 2018). Additionally, BCR-ABL1 dephosphorylation on Y245, a key residue for the kinase activity, together with the impairment of tyrosine phosphorylated residues stabilizing β-catenin, constitutes a fundamental issue for the disease progression (Della Peruta et al., 2010; Tomasello et al., 2020). Treatment with TKI and subsequent MMR (major molecular response) achievement was associated with a recovery of *PTPRG* expression, while *PTPRG* was still absent in patients who failed to achieve the MMR (Della Peruta et al., 2010; Vezzalini et al., 2017) suggesting that recovery of non-neoplastic hematopoiesis is associated to restoration of *PTPRG* expression. Recent flow cytometry analysis has confirmed this association between MMR and *PTPRG*-restored expression in CML patients after TKI-treatment (Figure 2; Table 2; Table 3) (Drube et al., 2018; Ismail et al., 2021).

**TABLE 2:** Cancerous RTKs interacting with PTPRG.

Proteins	Disease	PTPRG role	References
JAK2	CLL	Decrease integrin-mediated adhesion and increase apoptosis	(Mirenda et al., 2015; Montresor et al., 2021)
BTK	CLL	Decrease integrin-mediated adhesion and increase apoptosis	(Mirenda et al., 2015; Montresor et al., 2021)
BCR-ABL1	CML	Decrease cell proliferation	(Della Peruta et al., 2010; Vezzalini et al., 2017; Drube et al., 2018; Tomasello et al., 2020)
AXL	Ovarian Cancer	Decrease cell motility and cancer phenotype	(Antony et al., 2018; Zurzolo, 2018)
EGFR	NPC	Decrease cell proliferation, invasion and the angiogenesis processes	(Cheung et al., 2008; Cheung et al., 2015)
FGFR	Osteosarcoma	Decrease cell viability	Kostas et al. (2018)

**TABLE 3:** Hypothetical clinical role of PTPRG in cancers.

Disease	Predictive role	Prognostic effects	References
Chronic Myeloid Leukemia	CML patients with great response to therapy express high levels of PTPRG compared with levels at diagnosis	Increased levels of PTPRG mRNA have been found in patients who achieve the highest molecular response (MMR) after therapy compared to non-responders.	(Della Peruta et al., 2010; Vezzalini et al., 2017; Ismail et al., 2020; Ismail et al., 2021)
Lung Adenocarcinoma		Specific germline polymorphisms such as SNPs on PTPRG gene may influence the survival of patients with lung adenocarcinoma	Galvan et al. (2015)

### Carcinoma

Numerous reports indicate reduced *PTPRG* expression in epithelial-derived cancers. Data are available for several tissues and, more specifically, for lung (van Niekerk and Poels, 1999; Galvan et al., 2015), breast (Liu et al., 2016), gastric or esophageal squamous cell carcinoma (Wu et al., 2006; Lo et al., 2007), ovarian (van Niekerk and Poels, 1999), colorectal cancer (Wang et al., 2004; van Roon et al., 2011) and nasopharyngeal carcinoma (NPC) (Cheung et al., 2008; Cheung et al., 2015). A few studies addressed the role of PTPRG in nasopharyngeal carcinoma, where the extracellular matrix (ECM) seems to play a key role in cell-cycle progression. *PTPRG*-transfected NPC cells showed a significant cell-cycle arrest compared to the control particularly when these cell lines formed spheroids in 3D cultures, underlining the suppression induced by PTPRG through the interaction with ECM. Under these conditions, PTPRG reduces the phosphorylated form of Rb (active) producing the cell cycle G1-arrest through the regulation of cyclin D1 (Cheung et al., 2008). Subsequent investigations revealed an additional regulatory pathway involving PTPRG/EGFR/AKT in NPC (Yip et al., 2008). EGFR is regulated on Y1068 and Y1086 by PTPRG inducing downregulation of the PI3K/AKT pathway, also confirmed by the reduction of phosphorylation of several downstream substrates of AKT such as JNK, c-JUN and CREB (Figure 2 summarizes all PTPRG interactors). Remarkably, PTPRG suppresses invasive capacity on NPC cells while also limiting angiogenesis (Cheung et al., 2015). In epithelial ovarian cancer (EOC), the suppression of *OPCML*, a TSG recognized in several cancers, together with the overexpression of RPTK *AXL*, confer a worse overall survival (Sellar et al., 2003). In normal ovarian cells, Anthony J. et al. unearthed the chaperone interaction between OPCML protein and the active form of AXL kinase in the cholesterol-enriched lipid domains on cell membrane, in which PTPRG also resides. The proximity PTPRG mediated by OPCML to the AXL kinase produces an inhibitory effect on the AXL pathway and other RPTKs network, reducing the expression of transcription factors related to epithelial-mesenchymal transition (EMT) such as ZEB1 and related to cell motility such as Slug. Furthermore, the *PTPRG*-expressing cells were more sensitive to the AXL inhibitors, improving the therapeutic effect. In EOC, OPCML can be downregulated thereby preventing the tumor suppressor effect of PTPRG (Antony et al., 2018; Zurzolo, 2018). In addition to EOC, the lncRNA PTPRG-AS1 was found to be highly expressed in primary samples and cell lines compared to the normal counterparts. Indeed, it seems that PTPRG-AS1 may function as a sponge for miR-545-3p, which binds the 3’ UTR of the *HDAC4* gene causing both mRNA and protein repression. Interestingly, by interfering with PTPRG-AS1 expression, the tumorigenic capabilities of these cells have been considerably reduced both *in vitro* than in tumor xenograft model (Shi et al., 2020). The separation between PTPRG and AXL from one side (Antony et al., 2018) and the high expression of antisense lncRNA PTPRG-AS1 on the other (Shi et al., 2020), although with different pathways implication, suggest a fine and intricate regulation of PTPRG in the EOC. Similarly, in colorectal cancer the identification of somatic mutations suggests a complex scenario where *PTPRG* can be modified quantitatively (reduced expression driven by methylation/non-coding RNAs) and qualitatively (somatic mutations), a feature shared with the alteration of other classic TSGs in cancer development. (Capellini et al., 2021; Li et al., 2021). The case of Merkel Cell carcinoma is noteworthy, where integration of Merkel cell polyomavirus (MCV or MCPyV) to form different length fusions with intron 1 of the human *PTPRG* gene. Occurs virus incorporation was found associated in 80% of the cases and only 8% of controls (Feng et al., 2008), adding new scenario in the list of alterations occurring in the context of *PTPRG* gene and possibly contributing to the pathogenesis of this disease.

### Sarcoma

The observation that PTPRG and Fibroblast Growth Factor Receptors (FGFR1) interact and co-localize at the plasma membrane exhibiting a further model of regulation of PTPRG. Interestingly in U2OS sarcoma cells, PTPRG directly dephosphorylates the active FGFR1, connecting for the first time PTPRG to the development of sarcomas (Kostas et al., 2018). Indeed, FGFR overexpression and activating mutations were shown to play an important role in several types of sarcomas such as: osteosarcoma, rhabdomyosarcoma and soft tissue sarcoma (Taylor et al., 2009; Guagnano et al., 2012; Weekes et al., 2016; Zhou W.-Y. et al., 2016; Chudasama et al., 2017). Meanwhile FGFR-specific downstream signaling adaptor, FGFR substrate 2 (FRS2), is overexpressed in liposarcoma and renders these cells sensitive to FGFR inhibitors (Zhang et al., 2013; Hanes et al., 2016). In osteosarcoma the loss of PTPRG represent and advantage for cancer cells (a representative diagram is shown in Figure 2). Precisely, PTPRG regulates FGFR1 and it further appeared to impinge the efficiency of the TK-inhibitor on the FGFR kinase. This would represent a possible drug-resistance mechanism of cancer cells and the presence of PTPRG could reduce the effective concentration of the drug. Moreover, PTPRG could also modulate the activity of FGFR4 in rhabdomyosarcoma, indeed using siRNA against PTPRG in FGF-treated RH30 cell line increases phosphorylation of the receptor FGFR4 and downstream molecule such as PLCG1 (Phospholipase C-gamma 1) compared to the scramble control (Kostas et al., 2018). Another mechanism was recently found to occur in patients with osteosarcoma involving the long non-coding RNA PTPRG-AS1. Overexpression of PTPRG-AS1 may predict the poor prognosis of patients and may have a promoting effect on osteosarcoma cell metastasis being associated to increased migratory abilities of Saos-2 cells (Ge et al., 2021).

### Cancers of the Central Nervous System

Astrocytoma cell line U373-MG and primary astrocytes express *PTPRG* whose expression was found to be regulated by IL-1 or TNFα (Schumann et al., 1998; Lorenzetto et al., 2014). Analysis of formalin-fixed paraffin-embedded human tissues showed overexpression of PTPRG in astrocytoma cases with no or limited expression in their healthy counterparts (Vezzalini et al., 2007; Lorenzetto et al., 2014). In these tumor types, although *PTPRG* overexpression may be the manifestation of a putative oncogenic role, it might also be associated with the undifferentiated state of the neoplastic cells, as suggested by previous studies showing a role of this phosphatase in hematopoietic differentiation of murine embryonic cells and in neurite outgrowth (Shintani et al., 1997; Sorio et al., 1997). Notable, PTPRG has been involved in Wnt/β-catenin pathway involved in differentiation, cell migration and proliferation during embryogenesis and in adult tissues where a number of small molecules that can modulate it may have opposing effects depending on cell-type. Al-Harthi et al. reported that small molecules acting on colon epithelial cells do not have the same effect in astrocytes suggesting that different pathways involving β-catenin are active in CNS cells (Al-Harthi, 2012). However, the lack of knowledge regarding the function of *PTPRG* in this type of tumor does not allow a precise classification of its role.

A little bit features are available about the role of *PTPRG* in cancer affecting glial cells. The data report higher expression of the other member of R5 group *PTPRZ* in glioblastoma cells (Muller et al., 2003). Firstly, *PTPRZ*-knockdown has reduced some peculiar characteristics of the tumor such as proliferation, migration and growth as well as decreasing the expression of several transcription factors connected with the cancer-stemness, such as SOX2, OLIG2 and POU3F2. Secondly, the soluble portion of PTPRZ (sPTPRZ) constitutes a promising diagnostic biomarker present in the cerebrospinal fluid (CSF) which helps to identify different types of gliomas (Ulbricht et al., 2006; Fujikawa et al., 2016; Fujikawa et al., 2017; Yamanoi et al., 2020). In this tumor, both RPTPs members of R5 group were overexpressed possessing an oncogenic behavior. Noda group was able to synthesize specific inhibitors for the D1 domain of PTPRZ/PTPRG. NAZ2329 molecule increased the phosphorylation of some downstream targets of phosphatases (e.g. Y118-paxillin) limiting the proliferation and migration of some glioblastoma cell lines, confirming the effects produced by the knockout of the gene (Fujikawa et al., 2017). Nonetheless, data on these tumors are limited and further studies will be useful to confirm a specific oncogenic role for *PTPRG*.

## Conclusion

PTPRG is emerging as a multifunctional protein with multiple roles in healthy and disease tissues with still poorly characterized, emerging issues. As an example, strong PTPRG expression has been reported in endocrine cells of the gastrointestinal tract, pancreatic islets of Langerhans, adrenal medulla and thyroid, only associative evidence indicate a potential role in normal cell homeostasis/differentiation in this site (Brenachot et al., 2017) and that a dysregulated PTPRG could be involved the development of neuroendocrine tumors (Vezzalini et al., 2007). Numerous contributions that we have tried to summarize in this review have provided compelling evidence of a relevant role in cancers. More specifically, while large amounts of data are available for the hematopoietic system, much work done on solid tumors highlights what may be a “tip of the iceberg”. As we have seen, cancer cells implement different strategies to shutdown PTPRG, producing both the inhibition of expression and block the phosphatase activity. Altering the methylation state of the gene promoter appears to be one of the most commonly used systems in solid and non-solid cancer cells (Cheung et al., 2008; Della Peruta et al., 2010; van Roon et al., 2011; Chatterton et al., 2014; Stevenson et al., 2014; Xiao et al., 2014; Tomasello et al., 2020). Furthermore, with the advent of genomic technologies we have begun to understand additional post-translational regulatory mechanisms imposed by ncRNAs, such as miRNA and lncRNA. Surprisingly, the antisense RNA 1 of PTPRG (PTPRG-AS1) was found to be upregulated in different solid cancers representing a significant emerging predictor for tumor progression (Faghihi and Wahlestedt, 2009; Wang and Chang, 2011; Ge et al., 2021). Despite the fact that it has been clarified a suppressive role for *PTPRG* in various tumors, limited data suggesting an oncogenic role by the receptors PTPRZ/PTPRG in glioblastoma. Further studies will therefore be essential to explain this opposite pattern noted in CNS tumors (Fujikawa et al., 2016; Fujikawa et al., 2017; Yamanoi et al., 2020). Finally, besides to suggesting a potential pharmacological target that may be of interest in different types of malignancies, data currently available provide evidence to support a first possible clinical application as a “monitoring tool” for follow-up of CML patients. Considering the restoration of PTPRG expression in CML patients who reach the MMR (major molecular response) after TKI-treatment, as opposed to those who have not reached the MMR, can make the PTPRG a “tool” to monitor the recovery of normal hematopoiesis (Della Peruta et al., 2010; Vezzalini et al., 2017; Drube et al., 2018; Ismail et al., 2021). Clearly, a significant progress was done in the last decades since the initial cloning in 1993, with 143 publications mentioning *PTPRG* in PubMed database to date, with a trend to a year-by-year increase. However, much work is still be done to unveil the molecular details of this intriguing gene product, studies that are instrumental to exploit potential clinical application.

## Glossary

TSG: tumor suppressor gene
RPTKs: receptor protein tyrosine kinases
PTPs: protein tyrosine phosphatases
RPTPs: receptor protein tyrosine phosphatases
NRPTPs: non-receptor protein tyrosine phosphatases
PTPRG-AS1: PTPRG antisense 1
CNS: central nervous system
ncRNA: non coding RNA
RRE: ras-responsive element
LUAD: lung adenocarcinoma
NPC: nasopharyngeal carcinoma
ALL: acute lymphoblastic leukemia
CLL: chronic lymphocytic leukemia
CML: chronic myeloid leukemia
PLCG1: Phospholipase C-gamma 1
CTCF: CCCTC-binding factor
PTECs: renal proximal tubular epithelial cells
ERK: extracellular signal-regulated kinase
IkB: inhibitor of nuclear factor kappa B
JNK: c-Jun N-terminal kinase
MAPK: mitogen-activated protein kinase
BTK: Brutun’s tyrosine kinase
JAKs: janus tyrosine kinases
CXCR4: CXC motif chemokine receptor 4
BCRs: breakpoint cluster regions
ABL1: ABL proto-oncogene 1
TAT-ICD: cell-penetrating peptide/intracellular domain of PTPRG
CRKL: CRK like protein
STATs: signal transducer and activator of transcription
TKIs: tyrosine kinase inhibitors
EGFR: epidermal growth factor receptor
FGFR: fibroblast growth factor receptor
PI3Ks: phosphoinositide 3-kinases
AKT: protein kinase B
CREB: cAMP response element-binding protein
OPCML: opioid binding protein/cell adhesion molecule like
AXL: axl receptor tyrosine kinase
CSF: cerebrospinal fluid
